# Effects of soft pellet creep feed on pre-weaning and post-weaning performance and intestinal development in piglets

**DOI:** 10.5713/ajas.20.0034

**Published:** 2020-06-24

**Authors:** Hao Chen, Chunwei Wang, You Wang, Yilin Chen, Meng Wan, Jiadong Zhu, Aixia Zhu

**Affiliations:** 1 Hubei Key Laboratory of Animal Nutrition and Feed Science, Wuhan Polytechnic University, Wuhan 430023, China; 2 Hubei Collaborative Innovation Center for Animal Nutrition and Feed Safety, Wuhan 430023, China

**Keywords:** Suckling Piglet, Weaning Piglet, Creep Feed, Creep Feed Types, Growth Performance, Intestinal Development

## Abstract

**Objective:**

This study aimed to determine the effects of soft pellet creep feed (SPCF) on growth performance and intestinal development in piglets.

**Methods:**

A total of 18 sows and their litters of crossbred piglets (14±2 days, 3.73±0.72 kg) were assigned to one of three dietary groups receiving i) powder creep feed (PCF), ii) hard pellet creep feed (HPCF) or iii) SPCF during the pre-weaning period. After weaning, piglets were selected for continuous evaluation of the three diets on growth performance and intestinal health.

**Results:**

In the pre-weaning period, the average daily feed intake and average daily dry matter intake were significantly higher in the SPCF group than the HPCF group (p<0.05). In the post-weaning and entire experimental period, the different diets had no significant effect on growth performance. At 10 d after weaning, the serum glucose concentration was lower in the SPCF group (p<0.05) than the other groups; a higher (p<0.05) villus height and lower (p<0.05) crypt depth in the jejunum were also observed in the SPCF group than the other groups; Meanwhile, in the duodenum and jejunum, the SPCF group had a higher (p<0.05) villus height to crypt depth ratio than the other groups; Furthermore, the higher (p<0.05) threshold cycle values of lactic acid bacteria and lower (p<0.05) threshold cycle values of *Clostridium*, *Enterobacter* and *Escherichia coli* were also observed in the SPCF group, and the sucrase and maltase activity was higher (p<0.05) in the SPCF group than the other groups in duodenum and ileum.

**Conclusion:**

The SPCF improved pre-weaning feed intake and decreased the negative effects of weaning stress in the intestine in piglets.

## INTRODUCTION

During the later period of suckling, sow lactation decreases, and the weight and feed intake of piglets increases. Hence maternal milk cannot be used as the sole source of nutrients to meet piglets’ growth needs [1]. Moreover, piglets have relatively few diseases before the age of 2 weeks, but the longer the piglets stay with the sows, the more susceptible they are to diseases. Thus, many farms have begun to wean piglets early. However, the early weaning process is accompanied by a series of complex nutritional, physiological and behavioral changes, which can easily cause a condition known as early weanling stress syndrome of piglets [2]. Simultaneously, early weaning can lead to changes in morphology and function in piglets’ small intestines, such as villus atrophy and crypt hyperplasia, which decrease digestion and absorption, thus, causing a loss of appetite and continuous diarrhea, and possibly growth retardation and death [3]. To decrease the stress response caused by early weaning and to stimulate the growth potential of weaned piglets, creep feed is often used starting at 7 days of age. Creep feed consumption is believed to help initiate and promote the development of intestinal and digestive enzymes, which may help piglets use alternative food sources after milk is removed [4,5]. However, as the piglet diet changes from sow’s milk to hard and dry feed, the nutrients in raw plant material fed cannot be completely digested and absorbed in the intestines [6]. Therefore, lots of researches have been done to explore the nutritious creep feed, mainly focusing on feed additives, nutritional indicators and feed formula. However, although studies have demonstrated that the physical form of feed affects the growth performance of piglets [7], the effects of the physical form of feed on intestinal development in piglets have seldom been assessed. In addition, whether the feed hardness and moisture content affect piglet growth performance and intestinal health remain unclear.

Consequently, the current study was conducted to explore the potential advantages of new soft pellet creep feed (SPCF) on growth performance and intestinal function in piglets fed SPCF, powder creep feed (PCF) and hard pellet creep feed (HPCF).

## MATERIALS AND METHODS

### Preparation of creep feeds

The PCF was made of feedstuff by physical mixing, and HPCF was made with conventional pellet feed processing technology. Mix the powder and water at a certain ratio firstly, and then soft pellet feed was made by extrusion with high temperature. The detailed preparation of SPCF was as described in our laboratory’s European patent (No. 3251523). Randomly select several pellets in a uniform size and length, and then a texture analyzer (JWXL, Beijing, China) was used to measure the hardness of the pellets. An electric constant temperature drying oven (DHG-9141A, Shanghai, China) was used to determine the moisture content of the diets. The degree of starch gelatinization of the diets was determined with enzymatic hydrolysis as described by Medel et al [8], and a spectrophotometer (UV-1200, Shanghai, China) was used to measure the absorbance to calculate the degree of starch gelatinization.

### Animals and feeding

The experimental and animal handling procedures complied with the requirements of the Animal Care and Use Committee of Wuhan Polytechnic University. A total of 18 multiparous sows and their litters (Large White×Landrace) were used in this experiment. After 7 days of adaptation, according to the body weight (BW), back-fat thickness of sows and litter growth, the sows and piglets (14±2 days, 3.73±0.72 kg) were divided into three dietary groups receiving PCF, HPCF, or SPCF, with six replicate pens (10 pigs/pen) per group. The three creep feeds were formulated to have the same dietary composition, but different physical forms produced through different processing technologies. The experimental period lasted 17 days and was subdivided into two periods corresponding to the pre-weaning period (d 14 to 21) and post-weaning period (d 21 to 31), and weaning was performed in the morning on d 21. The piglets were housed in 2.20× 1.70 m pens with sows during pre-weaning period, and the sows were removed after piglets were weaned. Each pen was equipped with plastic slotted floor, a feeder and a nipple waterer to provide piglets with *ad libitum* access to feed and water. The nutrient content of the diets met the requirements for piglets suggested in NRC [9]. The formulas and chemical compositions of the diets used in this experiment are shown in Table 1.

### Piglet performance and sample collection procedures

The feed intake was recorded daily, and BW was measured with fasting for 12 h on d 14, d 21 and d 31 at 8:00 am. The average daily gain (ADG), average daily feed intake (ADFI), average daily dry matter intake (ADMI), the feed intake to BW gain ratio and the dry matter intake to BW gain ratio were calculated. In the morning of d 31, 18 piglets (1 per pen) were chosen for slaughter under anesthesia with an intravenous injection of sodium pentobarbital (50 mg/kg BW). The piglets selected from each pen had individual BW values close to the mean pen BW. Blood samples were taken from the jugular vein before euthanization. Blood samples were allowed to stand at room temperature and were then centrifuged at 3,000 r/min for 5 min to obtain serum. Serum was stored at −20°C. The animals were completely bled, and then the duodenum, jejunum and ileum were removed and flushed with ice-cold saline. A 3 cm long segment of intestine was harvested from the mid-duodenum, mid-jejunum and mid-ileum respectively, and stored in 4% paraformaldehyde for routine morphological measurements. Digested samples from the cecum fraction were collected in sterilized 2 mL conical tubes and immediately frozen with liquid nitrogen. Approximately 12 cm of the middle portions of the duodenum, jejunum and ileum were opened longitudinally and cleaned with ice-cold saline. Mucosal samples were collected by using a glass slide to scratch the connective tissue, snap-frozen in liquid nitrogen and then stored at −80°C until further analysis.

### Measurements

An automatic biochemical analyzer (RX Daytona TM, County Antrim, UK) was used, and colorimetric detection of total protein (TP), albumin (ALB), total cholesterol (TC), glucose (GLU), serum creatinine (SCREA), triglyceride (TG), calcium (Ca), and phosphorus (P) were measured. The activity of enzymes such as alkaline phosphatase (ALP), alanine aminotransferase (ALT), and aspartate aminotransferase (AST) were also measured. Fixed intestinal samples were prepared with standard paraffin embedding techniques. Three cross sections (4 μm thick) of each intestinal segment were stained with hematoxylin and eosin. Then the well-oriented, intact villi and their associated crypts from each segment were used to measure the villus height and crypt depth in a 10×10 mirror field of view (OLYMPSBX-41TF, Tokyo, Japan), and the villus height to crypt depth ratio was calculated. During the analysis, the activity of lactase, sucrase, and maltase was measured according to the method of Hou et al [10]. The activity of lactase, sucrase and maltase was assayed with a microplate reader (Spectra Max M5, Molecular Devices, Sunnyvale, CA, USA) by using a glucose kit (Nanjing Jiancheng Bio-engineering Institute, Nanjing, China) according to the manufacturer’s instructions.

To quantify the intestinal microflora, we determined the threshold cycle (Ct) values of total bacteria, lactic acid bacteria, *Enterococcus*, *Clostridium*, *Enterobacter*, and *Escherichia coli* in the cecum by using quantitative real-time polymerase chain reaction (ABI7500, Waltham, MA, USA). After digested samples were freeze-dried, genomic DNA was extracted and isolated with bead-beating with a QIAamp Fast DNA Stool Mini Kit (Qiagen, Germany). Microflora species-specific primers were designed and tested for specificity.

### Statistical analysis

All data were analyzed using one-way analysis of variance (ANOVA) of SPSS 20.0 software (SPSS Inc., Chicago, IL, USA). To analyze litter performance, litters were considered as experimental unit. The types of diet were considered as fixed effects, and parity and total born were considered as random effects. In order to determine the effect of diet types on intestinal development in weaned piglets, each piglet served as an experimental unit. The differences among group means were compared using Duncan multiple comparison based on the variance derived from ANOVA, and all data are expressed as means and standard error of the mean. The statistical significance level for all analyses was set at p≤0.05, and 0.05<p< 0.10 was considered to indicate a trend.

## RESULTS

### Characteristics of creep feeds

The degree of starch gelatinization, moisture content and hardness of the diets are shown in Table 2. The degree of starch gelatinization and moisture content of diets were significantly higher (p<0.05) in SPCF than the other two creep feeds. Simultaneously, the hardness was significantly lower (p<0.05) in SPCF than HPCF.

### Growth performance

In piglets 14 to 21 days of age, the ADFI and ADMI were significantly higher (p<0.05) in the SPCF group than the HPCF group and there was no significant difference in ADG among the groups (Table 3). In piglets 21 to 31 days of age, there were no significant difference in growth performance, and the feed intake to BW gain ratio and dry matter intake to BW gain ratio also did not differ among the groups. Throughout the entire experimental period, no significant differences in ADG, ADFI, and ADMI were observed among the groups.

### Feed intake of piglets on the days before and after weaning

As shown in Table 4, at d 2, d 1 before weaning and d 2 after weaning, the feed intake was significantly higher (p<0.05) in the SPCF group than the PCF and HPCF group. Meanwhile, at d 2 before weaning, the dry matter intake tended to be higher (p = 0.06) in the SPCF group than the HPCF group. At d 1 before weaning, the SPCF groups also had higher (p< 0.05) dry matter intake than the other groups. At the day of weaning, there was no significant difference in feed intake and dry matter intake among the groups.

### Serum biochemical indexes

No differences were observed in the serum concentrations of TP, ALB, AST, ALT, TC, TG, Ca, P, or SCREA among the groups (Table 5). However, the serum concentration of GLU in the SPCF group was lower (p<0.05) than that in the other groups, and the SPCF group tended to have lower (p = 0.06) serum concentrations of ALP than the PCF group.

### Influence of different types of creep feed on gut physiology and microbiology

**Figure 1:**
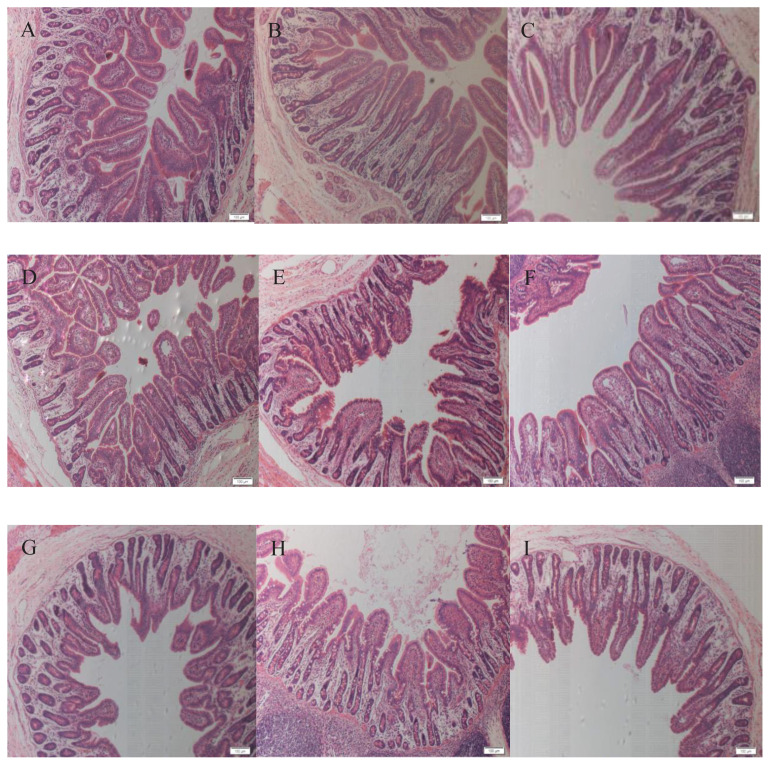
A 3 cm long segment of intestine was harvested from the mid-duodenum, mid-jejunum and mid-ileum of piglets at 10 d after weaning, and stored in 4% paraformaldehyde for routine morphological measurements. Fixed intestinal samples were prepared with standard paraffin embedding techniques, and hematoxylin and eosin stained slides were prepared. Then the villus height and crypt depth were observed in a 10×10 mirror field of view. A to C: the morphological structure of duodenum; D to F: the morphological structure of jejunum; G to I: the morphological structure of ileum. A, D, G: piglets received powder creep feed; B, E, H: piglets received hard pellet creep feed; C, F, I: piglets received soft pellet creep feed.

Part of hematoxylin and eosin staining results are shown in Figure 1. In the duodenum, greater (p<0.05) villus height and crypt depth were observed in the HPCF group than the PCF and SPCF groups, and the villus height was greater (p< 0.05) in the SPCF group than the PCF group (Table 6). Furthermore, there was no significant difference in crypt depth between the PCF and SPCF groups. Therefore, the villus height to crypt depth ratio was higher (p<0.05) in the SPCF group than the PCF and HPCF groups. In the jejunum, the villus height was greater (p<0.05) in the SPCF group than the other groups. Moreover, lower (p<0.05) crypt depth was observed in the SPCF group than the other groups. Thus, the villus height to crypt depth ratio was higher (p<0.05) in the SPCF group than the PCF and HPCF groups. In the ileum, no significant difference was observed in villus height among the groups. However, lower (p<0.05) crypt depth was observed in the SPCF and PCF groups than the HPCF group. Moreover, the villus height to crypt depth ratio was higher (p<0.05) in the SPCF and PCF groups than the HPCF group.

The real time polymerase chain reaction Ct values of mi croflora in the cecum are presented in Table 7. The Ct values of total bacterial populations were unaffected in the different groups, and the SPCF group showed greater (p<0.05) Ct values for lactic acid bacteria than the PCF and HPCF groups. The SPCF group showed lower (p<0.05) Ct values for *Enterococcus* than those in the HPCF group, and the Ct values for *Clostridium* were lower (p<0.05) in the SPCF group than the other groups. Similarly, the Ct values for *Escherichia coli* and *Enterobacter* were lower (p<0.05) in the SPCF group than the PCF and HPCF groups.

### Influence of different types of creep feed on intestinal mucosa disaccharidase activity

The specific activity of sucrase, maltase and lactase in the duodenum, jejunum and ileum is shown in Table 8. The SPCF group had greater (p<0.05) maltase activity in the duodenum, jejunum and ileum, and higher (p<0.05) sucrase activity in the duodenum and ileum, than the other groups. In contrast, the lactase activity in the duodenum was lower (p<0.05) in the SPCF group than the two other groups, but there were no significant differences in lactase activity in the jejunum and ileum among groups.

## DISCUSSION

The suckling period is a key time for the growth performance and the intestinal development of piglets, during which high intake of nutrients is required. Providing creep feed before weaning can significantly increase the feed intake of piglets after weaning and also prevent growth inhibition [11,12]. Kim et al [13] have reported that the physical form of diets affects the performance of early weaned piglets.

In present study, the weight gain was higher than the feed intake of piglets in all groups from d 14 to d 21, which may due to piglets favored sow’s milk before weaning. The effect of sow’s milk on piglet growth performance was greater than that of creep feed. These findings were similar to those reported by Okai et al [14], in which piglets were found to acquire most of their energy from consumption of maternal milk, but only a small amount from creep feed in the third or fourth week of age. Pluske et al [15] have reported that providing water in addition to food in a single-space feeder might stimulate voluntary food intake further. Moreover, Barber et al [16] have also shown that piglets prefer to eat food when water is available. In the current study, the SPCF group had the higher ADFI and ADMI than the HPCF group during pre-weaning period. These findings may have occurred because SPCF with higher moisture had greater starch gelatinization and lower hardness than the other creep feeds, thereby resulting in greater palatability of feed. Similarly, Partridge et al [17] have reported that when water is added to a dry diet in a 1:1 ratio to form a slurry, there is significant increase in feed intake, thus supporting the observations in the current study and also indicating that increasing the moisture content of feed can improve the attractiveness of feed and consequently feed intake in piglets. In the present study, after weaning, all groups had no significant difference in ADG, the feed intake to BW gain ratio and the dry matter intake to BW gain ratio. Therefore, these results indicate that providing SPCF during the suckling period helps to improve the feed intake of piglets, but has no significant effect on the growth of piglets after weaning. In addition, compared with the piglets after weaning in common piggery farms, all groups had lower ADG and higher ratio of feed intake to BW gain in the current study. These results may be related to the experimental environment and the stress brought to the piglets when piglets were fed, and the higher ratio of feed intake to BW gain may be a result of wasting feed when piglets rushed to eat.

Feed intake is important for piglet growth performance in the days before and after weaning, and post-weaning feed intake is also closely associated with that of pre-weaning. Creep feed intake during lactation has been shown to stimulate early post-weaning feed intake [11,12]. In the present study, the higher feed intake in the SPCF group than the other groups at d 2 before weaning. Meanwhile, both the feed intake and dry matter intake of the SPCF group was higher than that of the HPCF and PCF groups at d 1 before weaning. Moreover, at d 2 after weaning, the SPCF group still had higher feed intake than the other groups. These results were similar to those of Cranwell et al [18], who have demonstrated that pre-weaning creep feed intake stimulates further post-weaning feed intake and decreases the time to consumption of post-weaning diets. Meanwhile, these results also show that SPCF feed had better attractive effect than the other diets. In addition, in the current study, compared with the feed intake at 2 days before weaning, there was no decrease in feed intake on the day of weaning in each group. Thus, indicating that feeding creep feed during lactation effectively prevents the decrease in piglet feed intake caused by weaning. Moreover, compared with the feed intake at the 2 days after weaning, all groups had lower feed intake at the day of weaning, which may due to piglets suffered much weanling stress at the day of weaning.

The increase in serum GLU concentrations may have been a result of social stress due to mixing of unfamiliar animals [19]; when suffering greater stress, the piglets often have higher serum GLU levels. In addition, an increase in ALP activity often reflect the dysfunction of heart or muscle tissues [20]. In current study, the SPCF group had the lowest serum GLU levels, meanwhile, the serum GLU concentrations were within the normal reference range (50 to 79 mg/dL; Marshfield Clinic Laboratories Veterinary Division) [21]. Therefore, indicating that piglets fed SPCF tend to have lower weaning stress. Furthermore, the SPCF group tended to have lower serum ALP levels than the PCF group, which also indicates that piglets fed SPCF tend to show less dysfunction of muscle tissues than those fed PCF after weaning.

In piglet weaning, the piglet diet gradually shifts from maternal milk to complex artificial feed, which is generally supplied in a solid form. At this stage, effects are observed in piglet gastrointestinal tract function, usually manifested as changes in gastrointestinal morphology, and the intestinal villus height and absorption capacity of piglets decrease [22]. Similarly, Heo et al [1] have also found that drastic changes occur in the gastrointestinal tract of piglets after weaning. When piglets adapt to solid feeds, their intestinal villi become thicker and shorter, and the depth of the crypts deepens. At weaning, the piglet small intestine usually shows a decrease in villus height and an increase in crypt depth, which are linked to a decrease in absorptive capacity. This condition may result in malabsorption, increased intestinal fermentation, post-weaning diarrhea and decreased feed intake [15]. In present study, the greatest villus height and the lowest crypt depth were observed in the SPCF group in the jejunum, and the SPCF group also had lower crypt depth than the HPCF group in the ileum. These results were consistent with those of Pluske et al [23], who have suggested that wet feeding can decrease the intestinal villus atrophy and crypt depth caused by weaning. Deprez et al [24] have also compared the effects of wet and dry feeding on the villus height and crypt depth in the same piglet diet and reached a similar conclusion in which dry feeding causes the intestinal villi to shrink and the crypt to become deeper. If the ratio of villus height to crypt depth is compared, the differences between the two feeding methods become clear. Softer and higher moisture content of creep feed resulted in less damage to the piglet intestine, and showed benefits in intestinal development. The ratio of intestinal villus height to crypt depth determines the ability of piglets to digest and absorb nutrients: greater values indicate a greater contact area between the gut and nutrients, absorption area, and utilization of nutrients [25]. In current study, the SPCF group not only had the highest ratio of villus height to crypt depth in the duodenum and jejunum, but also had higher ratio of villus height to crypt depth than the HPCF group in the ileum. These results indicated that SPCF, compared with the other two creep feeds, was associated with better intestinal development and digestion of nutrients.

After piglets are weaned, the intestinal flora changes: the concentrations of pathogenic bacteria such as *Escherichia coli* increase, and those of beneficial bacteria, such as lactic acid bacteria, decrease [26]. In current study, the SPCF group had fewer *Escherichia coli*, *Clostridium*, and *Enterobacter* in the cecal contents than the other groups, and the number of lactic acid bacteria was also significantly higher than that in the other two groups. These results indicated that SPCF promoted the growth of probiotics and inhibited the growth of pernicious bacteria.

Early weaning of piglets not only affects intestinal mor phology, but also leads to changes in intestinal enzyme activity in the intestine [23]. Several studies have also reported that solid feed stimulates acid production and digestive enzyme activity [18,27]. Most digestion and absorption of nutrients occurs in the small intestine in piglets, where disaccharidase is an important digestive enzyme. Apart from changes in endogenous disaccharidase, feeding methods [28], diets [29] and hormone treatment can affect enzyme activity. Similarly, in this study, the disaccharidase activity in the small intestine mucosa differed when piglets were fed different diets. In the current study, the SPCF group not only had the highest sucrase and maltase activity in the duodenum and ileum, but also had highest maltase activity in the jejunum. Meanwhile, no significant differences were observed among the groups in lactase activity in the jejunum and ileum. In addition, the lower lactase activity was observed in SPCF group than that of other groups in the duodenum, and the reason needs to be explored further. In general, these results indicated that diet type significantly affects the activity of disaccharidase, and SPCF with higher moisture improves sucrase and maltase activity and helps to increase the utilization of carbohydrates.

In conclusion, SPCF improved the feed intake of piglets during suckling period, and effectively promoted intestinal development and decreased the negative effects of weaning stress on the piglet intestinal tract.

## Figures and Tables

**Table 1:** Formula and chemical composition of creep diets in the experiment

Items	Content
Ingredients (%)	
Corn	40.00
Soybean meal	25.70
Fish meal	5.00
Whey powder	7.50
Plasma protein powder	5.00
Glucose	5.00
Sugar	4.00
Soybean oil	5.00
Calcium phosphate dibasic	1.00
Limestone	0.50
Salt	0.30
Vitamin and mineral premix1)	1.00
Sum	100.00
Nutrient composition	
Metabolizable energy2) (MJ/kg)	14.51
Crude protein3) (%)	22.49
Calcium3) (%)	0.84
Total phosphorus3) (%)	0.68
Lysine3) (%)	1.40
Methionine3) (%)	0.36

1) The premix provided the following amounts per kilogram of complete diet: Supplied the following per kilogram of complete diet: 8,500 IU of vitamin A; 1,000 IU of vitamin D_3_; 10 IU of vitamin E; 1.0 mg of vitamin K_3_; 3.6 mg of vitamin B_2_; 1.5 mg of vitamin B_6_; 0.25 mg of biotin; 0.6 mg of folic acid; 10 mg of D-pantothenic acid; 10 mg of niacin; 1.2 g of choline chloride; 100 mg of Fe; 10 mg of Cu; 20 mg of Mn; 100 mg of Zn; 0.75 mg of Co; 0.2 mg of I; 0.3 mg of Se.

2) Calculated.

3) Analyzed.

**Table 2:** Characteristics of the experimental diets

Items	Group			SEM	p-value

	Powder creep feed	Hard pellet creep feed	Soft pellet creep feed		
Moisture content (%)	10.07^A^	12.76^B^	26.97^C^	0.17	<0.01
Starch gelatinization (%)	5.65^A^	45.08^B^	86.40^C^	0.90	<0.01
Hardness (g)	-1)	2,690^B^	505^A^	69	<0.01

SEM, standard error of the mean.

1) The hardness of powder creep feed can not be determined. “-” means no data.

A–C Means in the same row with different superscript letters were significantly different (p≤0.05).

**Table 3:** Effect of different physical forms of creep feed on growth performance of piglets

Items	Group1)			SEM	p-value

	PCF	HPCF	SPCF		
BW (kg)					
Day 14	3.84	3.63	3.71	0.31	0.90
Day 21	4.58	4.74	4.84	0.32	0.84
Day 31	5.40	5.57	5.75	0.35	0.79
ADG (g)					
Day 14 to 21	127	159	160	23.15	0.54
Day 21 to 31	82	77	88	9.18	0.74
Overall	92	114	119	15.12	0.42
ADFI (g)					
Day 14 to 21	53^AB^	31^A^	73^B^	7.74	<0.01
Day 21 to 31	188	162	198	26.67	0.62
Overall	132	108	146	16.61	0.29
Feed/gain ratio					
Day 21 to 31	2.25	2.15	2.32	0.25	0.89
ADMI (g)					
Day 14 to 21	47^AB^	27^A^	53^B^	6.17	0.02
Day 21 to 31	169	141	145	23.31	0.67
Overall	119	94	107	14.72	0.52
Dry matter/gain ratio					
Day 21 to 31	2.03	1.88	1.70	0.21	0.55

SEM, standard error of the mean; BW, body weight; ADG, average daily gain; ADFI, average daily feed intake; ADMI, average daily dry matter intake.

1) PCF, piglets received powder creep feed; HPCF, piglets received hard pellet creep feed; SPCF, piglets received soft pellet creep feed.

A,B Means in the same row with different superscript letters were significantly different (p≤0.05).

**Table 4:** Effect of different physical forms of creep feed on feed intake of piglets before and after weaning

Items	Group1)			SEM	p-value

	PCF	HPCF	SPCF		
Feed intake (g)					
Day 2 before weaning	33.9^A^	31.3^A^	68.1^B^	8.04	<0.01
Day 1 before weaning	36.8^A^	28.0^A^	82.1^B^	10.75	<0.01
At weaning	68.1	36.9	97.0	20.30	0.15
Day 1 after weaning	148.8	133.0	164.5	25.85	0.70
Day 2 after weaning	127.1^A^	146.2^A^	199.1^B^	14.73	0.01
Dry matter intake (g)					
Day 2 before weaning	30.5^ab^	27.3^a^	49.7^b^	6.56	0.06
Day 1 before weaning	33.1^A^	24.4^A^	59.9^B^	8.45	0.02
At weaning	61.3	32.2	70.8	17.08	0.28
Day 1 after weaning	133.8	116.0	120.1	21.96	0.84
Day 2 after weaning	114.3	127.6	145.4	11.84	0.21

SEM, standard error of the mean.

1) PCF, piglets received powder creep feed; HPCF, piglets received hard pellet creep feed; SPCF, piglets received soft pellet creep feed.

A,B Means in the same row with different superscript letters were significantly different (p≤0.05).

a,b Means in the same row with different superscript letters tended to be different (0.05<p<0.10).

**Table 5:** Effect of different physical forms of creep feed on serum biochemical parameters of piglets at 10 d after weaning

Items	Group1)			SEM	p-value

	PCF	HPCF	SPCF		
TP (g/L)	33.9	33.7	34.1	2.20	0.99
ALB (g/L)	22.0	23.9	22.6	1.30	0.60
AST (U/L)	43.5	39.2	68.8	10.26	0.12
ALT (U/L)	30.3	30.7	34.5	3.62	0.67
GLU (mmol/L)	114.1^B^	112.3^B^	75.6^A^	10.96	0.04
TC (mmol/L)	56.6	60.6	64.9	5.94	0.63
TG (mmol/L)	23.1	23.4	36.6	5.81	0.20
ALP (U/L)	828.7^b^	668.0^ab^	548.8^a^	75.10	0.06
Ca (mmol/L)	8.7	9.0	8.8	0.35	0.88
P (mmol/L)	5.6	7.2	7.1	0.55	0.11
SCREA (μmol/L)	72.8	70.7	67.3	8.46	0.90

SEM, standard error of the mean; TP, total protein; ALB, albumin; AST, aspartate aminotransferase; ALT, alanine aminotransferase; GLU, glucose; TC, total cholesterol; TG, triglyceride; ALP, phosphatase; Ca, calcium; P, phosphorus; SCREA, serum creatinine.

1) PCF, piglets received powder creep feed; HPCF, piglets received hard pellet creep feed; SPCF, piglets received soft pellet creep feed.

A,B Means in the same row with different superscript letters were significantly different (p≤0.05).

a,b Means in the same row with different superscript letters tended to be different (0.05<p<0.10).

**Table 6:** Effect of different physical forms of creep feed on intestinal morphology in piglets at 10 d after weaning

Items	Group^2)^			SEM	p-value

	PCF	HPCF	SPCF		
Duodenum					
Villus height (μm)	519^A^	715^C^	638^B^	18.79	<0.01
Crypt depth (μm)	362^A^	471^B^	367^A^	15.78	<0.01
Villus/crypt ratio	1.56^A^	1.60^A^	1.86^B^	0.07	<0.01
Jejunum					
Villus height (μm)	192^A^	219^B^	252^C^	6.05	<0.01
Crypt depth (μm)	178^B^	188^B^	157^A^	6.25	<0.01
Villus/crypt ratio	1.17^A^	1.30^A^	1.79^B^	0.07	<0.01
Ileum					
Villus height (μm)	251	240	257	7.14	0.24
Crypt depth (μm)	156^A^	174^B^	151^A^	5.49	<0.01
Villus/crypt ratio	1.72^B^	1.45^A^	1.86^B^	0.07	<0.01

SEM, standard error of the mean.

1) PCF, piglets received powder creep feed; HPCF, piglets received hard pellet creep feed; SPCF, piglets received soft pellet creep feed.

A–C Means in the same row with different superscript letters were significantly different (p≤0.05).

**Table 7:** Effect of different physical forms of creep feed on the microflora population in the cecum of piglets at 10 d after weaning

Items	Group1)			SEM	p-value

	PCF	HPCF	SPCF		
Total bacteria2)	1.00	1.13	1.02	0.06	0.32
Lactic acid bacteria	1.00^A^	0.92^A^	1.25^B^	0.07	0.02
*Enterococcus*	1.00^A^	1.35^C^	1.13^B^	0.04	<0.01
*Clostridium*	1.00^B^	1.04^B^	0.53^A^	0.08	<0.01
*Escherichia coli*	1.00^C^	0.64^B^	0.29^A^	0.06	<0.01
*Enterobacter*	1.00^C^	0.27^B^	0.12^A^	0.03	<0.01

1) PCF, piglets received powder creep feed; HPCF, piglets received hard pellet creep feed; SPCF, piglets received soft pellet creep feed.

2) Microflora populations are represented with the Ct value from the real time polymerase chain reaction.

SEM, standard error of the mean.

A–C Means in the same row with different superscript letters were significantly different (p≤0.05).

**Table 8:** Effect of different physical forms of creep feed on activities of intestinal disaccharidase in piglets at 10 d after weaning (U/mg)

Items	Group1)			SEM	p-value

	PCF	HPCF	SPCF		
Duodenum					
Sucrase	138.7^A^	134.7^A^	157.9^B^	4.80	<0.01
Maltase	6.7^A^	15.3^B^	20.2^C^	1.47	<0.01
Lactase	44.6^B^	41.3^B^	23.7^A^	3.77	<0.01
Jejunum					
Sucrase	140.7^B^	94.1^A^	142.5^B^	2.07	<0.01
Maltase	19.7^B^	17.5^A^	30.4^C^	0.29	<0.01
Lactase	17.7	18.9	19.8	0.84	0.24
Ileum					
Sucrase	27.3^A^	37.7^B^	65.7^C^	3.30	<0.01
Maltase	40.8^A^	34.8^A^	76.7^B^	2.80	<0.01
Lactase	10.2	10.2	10.0	0.13	0.27

SEM, standard error of the mean.

1) PCF, piglets received powder creep feed; HPCF, piglets received hard pellet creep feed; SPCF, piglets received soft pellet creep feed.

A–C Means in the same row with different superscript letters were significantly different (p≤0.05).
